# Hearing Impairments as an Overlooked Condition in Kidney Transplant Recipients

**DOI:** 10.3389/ti.2022.10198

**Published:** 2022-04-12

**Authors:** Melis Simsir, Muhammed Gazi Yildiz, Murat Karatas, Abdullah Dalgic, Ilyas Ozturk, Erhan Tatar, Necmi Eren, Ertugrul Erken, Ozkan Gungor, Orcun Altunoren

**Affiliations:** ^1^ Department of Internal Medicine, Faculty of Medicine, Kahramanmaras Sutcu Imam University, Kahramanmaras, Turkey; ^2^ Department of Otolaryngology Head and Neck Surgery, Faculty of Medicine, Kahramanmaras Sutcu Imam University, Kahramanmaras, Turkey; ^3^ Department of General Surgery, Izmir Bozyaka Education and Research Hospital, Izmir, Turkey; ^4^ Department of Otolaryngology Head and Neck Surgery, Izmir Bozyaka Education and Research Hospital, Izmir, Turkey; ^5^ Department of Nephrology, Faculty of Medicine, Kahramanmaras Sutcu Imam University, Kahramanmaras, Turkey; ^6^ Department of Nephrology, Izmir Bozyaka Education and Research Hospital, Izmir, Turkey; ^7^ Department of Nephrology, Faculty of Medicine, Kocaeli University, Kocaeli, Turkey

**Keywords:** kidney transplantation, extended high-frequency audiometry, hearing impairment, hemodialysis, immunsuppressants

## Abstract

It is not known whether hearing disorders improves with kidney transplantation. One of the neurotoxic effects of immunosuppressive drugs may be unrecognized hearing loss. In this study, our aim was to evaluate the hearing disorders in kidney transplant patients. Hearing problems in 46 kidney transplant patients [eGFR ≥ 60 ml/min/1.73 m^2^ (30 Tacrolimus, 16 mTOR inhibitor users)], 23 hemodialysis patients, and 20 healthy controls were evaluated with a questionnaire and high-frequency audiometry. More than half (58.7%) of the transplant patients had at least one hearing problem. Hearing loss was observed in 50%, 60.9% and 76.1% of the transplant patients at 8,000, 16,000 and 20,000 Hz. Hearing thresholds of transplant and hemodialysis patients increased from 4,000 to 20,000 Hz and was higher than that of controls. Hearing thresholds were higher at 1,000–2,000 Hz in patients using tacrolimus and at 16,000–20,000 Hz in patients using mTOR inhibitor. No correlation was found between hearing threshold and blood tacrolimus or mTOR inhibitor levels. Most kidney transplant and hemodialysis patients have hearing loss at higher frequencies than medium frequencies. Hearing loss in chronic kidney patients is likely to be permanent and kidney transplantation may not improve hearing problems. Hearing problems may be more pronounced at medium frequencies in patients receiving tacrolimus but at higher frequencies in patients receiving mTOR inhibitors.

## Graphical Abstract

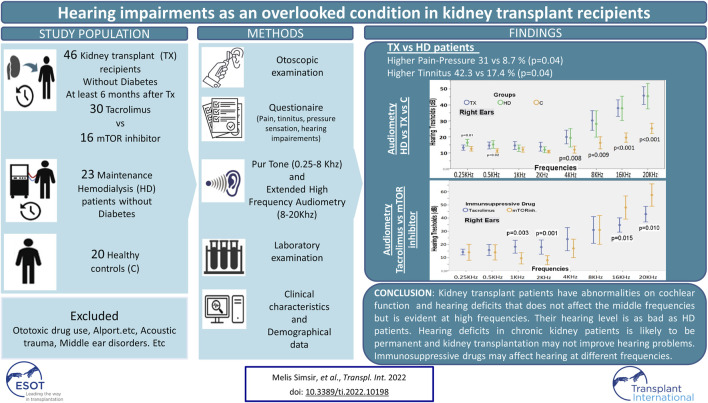

## Introduction

Kidney transplantation is the most preferred treatment method for end-stage renal disease. According to the World Health Organization data, in 2019, 100,097 kidney transplants were performed all over the world (1). Kidney transplant patients take lifelong immunosuppressive drugs, which have many side effects. Currently, many transplant centres use tacrolimus as a calcineurin inhibitor (CNI) in their immunosuppressive regimen while mTOR inhibitors are used much less frequently (2,3). Calcineurin inhibitors generally have a similar side effect profile, with the most important one being neurotoxicity (4). Tacrolimus is slightly more neurotoxic than Cyclosporine (CsA) (4,5). Although neurotoxicity is most commonly seen in the form of tremors, more serious conditions such as epileptic seizures and confusion may also occur (6).

We have observed that some kidney transplant patients, albeit very few, experience hearing problems after transplantation. Advanced age, diabetes, ototoxic drug use, and uremia can partially explain this situation; it is possible that the immunosuppressive drugs used, especially tacrolimus, may also have ototoxic effects. It is accepted that mTOR inhibitors do not have neurotoxicity (7-9). There are only a few studies in the literature investigating hearing problems in kidney transplant patients. Moreover, there are no studies showing whether there is a relationship between the type of immunosuppressive drug used and hearing problems. Hearing tests are usually conducted in the 125–8000 Hz. The 9000–20000 Hz range is called Extended High-Frequency Audiometry (EHFA), and it is important tool in detecting hearing loss that starts at high frequencies and progresses to low frequencies, due to reasons such as aging and toxic causes (10).

In this study, our primary aim was to determine any hearing problems in kidney transplant patients in kidney transplant patients using questionnaire and EHFA. Our second goal was to determine whether there was a relationship between the hearing problem, if any, and the type of immunosuppressive drug used.

## Patients and Methods

This cross-sectional case-control study was conducted at the Department of Nephrology and Department of Ear-Nose, and Throat Clinics of Kahramanmaras Sutcu Imam University Medical Faculty Hospital, and Izmir Bozyaka Training and Research Hospital. A total of 89 patients; 46 kidney transplant recipients (TX group), 23 hemodialysis patients (HD group) and 20 healthy controls (C group) were included in the study. Ethical approval was obtained from the local ethics committee of Kahramanmaras Sutcu Imam University (date: September 09, 2020, session no.2020/17, decision no.18). Written informed consent was obtained from all patients.

### Inclusion Criteria

Nondiabetic kidney transplant recipients with an estimated glomerular filtration rate (eGFR) > 60 ml/min/1.73 m^2^ (calculated with the MDRD formula), between the ages of 18–50 years, that have passed at least 6 months after kidney transplant, and have been using tacrolimus or an mTOR inhibitor (Everolimus) in their immunosuppressive regimen were included in the TX group. Nondiabetic patients aged 18–50 years and that have received maintenance hemodialysis treatment three sessions/week for at least 6 months were included in the HD group. Healthy subjects who matched with kidney transplant patients for age and gender distribution were included in the control group.

### Exclusion Criteria

Patients under the age of 18 and over 50, were diagnosed with Alport syndrome, had a known or newly developed diabetes mellitus (DM), have used ototoxic drugs within the last 3 months (furosemide, torsemide, aminoglycoside antibiotics, erythromycin, vancomycin, etc.), had a history of hereditary or acquired hearing loss problems due to several reasons (acoustic trauma, genetic syndromes with hearing loss, neurological-psychiatric problems, those with recurrent upper respiratory tract infection, tympanic membrane and middle ear pathology in otoscopic examination, Meniere’s disease, Cogan Syndrome, Costen Syndrome, etc.), have had ear trauma or surgery, have intracranial pathology that may cause hearing loss, have malignancy and been receiving chemotherapy, and those in whom the time elapsed since the start of dialysis or after kidney transplant was less than 6 months, HD patients whose Kt/V value was less than 1.2 within the last 3 months, kidney transplant patients with eGFR < 60 ml/min/1.73 m^2^, kidney transplant patients using regimens that do not contain tacrolimus or mTOR inhibitors were not included in the study.

### Obtaining of Demographic and Laboratory Data

Data such as patients’ age, gender, presence of comorbidity (hypertension or coronary artery disease,etc), the etiology of chronic kidney disease (CKD), the number of years they have been receiving HD treatment, duration of RRT (renal replacement therapy) before transplantation, the time elapsed after kidney transplant, and the immunosuppressive drugs used were obtained from their medical records. Systolic and diastolic blood pressures (SBP and DBP), height and weight were measured before the midweek dialysis session for HD patients and during the examination for TX and C groups. Body mass index (BMI) was calculated by dividing body weight in kilograms divided by square of height in meters.

Fasting blood glucose (FBG), blood urea nitrogen (BUN), creatinine (Cr), sodium (Na), potassium (K), calcium (Ca), phosphorus (P), LDL cholesterol, triglyceride (TG), uric acid (UA), serum albumin levels were measured with the standard methods. A blood sample of the HD patients was taken just before the midweek dialysis session. The patients’ Kt/V values and urea reduction ratios (URR) were retrieved from their medical records, and the arithmetic average of the last 3 months was calculated. The blood of TX patients was taken during outpatient control and tacrolimus or mTOR inhibitor blood levels (C0) were measured.

### Audiometric Measurements and Ear Examination

Audiometric measurements and the examinations of the external and middle ear and the throat were performed by a single otolaryngology specialist in each centre. Lavage and aspiration were performed on patients who required plug aspiration. All audiological evaluations were performed in a standard double-wall, soundproof booth (IAC Acoustics, Naperville, IL, United States). Airway hearing tresholds at 250, 500, 1,000, 2,000, 4,000 and 8,000 Hz frequencies using the Telephonics TDH 39P headphones (Telephonics Corp., Farmingdale, NY, United States) with the Interacoustics AC-40 audiometer (Interacoustics, Middelfart, Denmark), bone conduction hearing thresholds at the 500, 1,000, 2,000 and 4,000 Hz frequencies using the Rodioear B-71 bone transducer (RadioEar, Middelfart, Denmark), and high frequency airway hearing thresholds (>8,000 Hz) using Harward HR H903 headphones were determined. The mean and standard deviation of air and bone conduction thresholds were calculated for each frequency for all patients. The actual hearing levels were determined by masking in patients with a hearing level difference of more than 40 dB between both ears and an air-bone conduction difference of >10 dB. Apart from audiometric examinations, immitance measurements were made using Interacoustics AZ26 and AT235h clinical tympanometry devices. Middle ear pressur and ipsilateral and contralateral acoustic reflex thresholds of all participants were evaluated. In addition, Speech Reception Treshold (SRT), Speech Discrimination (SD) tests were performed on all patients.

### Survey Data on Hearing

To define hearing problems, TX and HD patients were asked the following survey questions appropriate to the patient’s group.

1. Did you have a hearing problem before the transplant?
2. Did your hearing decrease after the transplant?
3. Do you feel the need for a hearing aid?
4. Do you have any ear pain or a feeling of pressure in the ear?
5. Do you have ringing in the ear (Tinnitus)?
6. Do you have dizziness?
7. Did you experience sudden hearing loss after the transplant?
8. Have you had ear surgery?
9. Have You had an Ear/Head Trauma?

### Statistical Analysis

The power analysis of the study was performed with the G*Power 3.1.9.7 for Windows (11) software, and it was predicted that with the inclusion of 20 patients in each group, the alpha error would be 0.005 and the power of the study would be 99%. The SPSS v16.0 was used for statistical analysis. Data obtained by measurement were expressed as mean ± SD, and categorical data obtained by counting were expressed as percentages or ratios. The distribution characteristics of the data were evaluated with the Shapiro Wilk test. The Kruskal Wallis analysis of variance was used to compare the data obtained by measurements in the TX, HD and C groups. A *p* value of less than 0.05 was considered statistically significant. Bonferonni correction was applied to evaluate which group caused the difference; the groups were compared in pairs, and a *p* value of less than 0.016 was considered significant. The categorical variables were evaluated with the Chi-square test. The Mann-Whitney U and chi-square test were used to compare patients using tacrolimus or an mTOR inhibitor according to data type. A *p* value of less than 0.05 was considered significant.

## Results

### Survey Results for Hearing Problems

**FIGURE 1:**
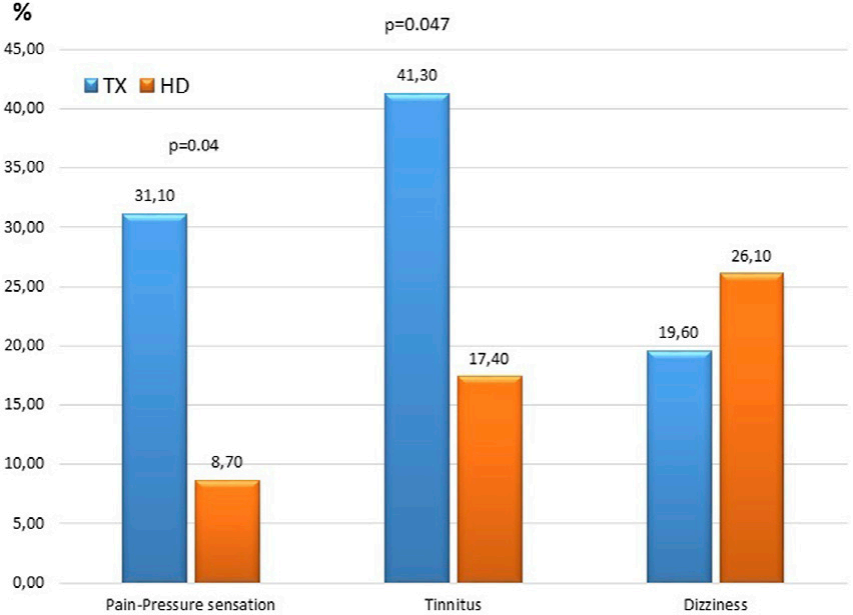
Hearing problems in the HD and TX patients according to the survey results.

More than half (58.7%) of the TX patients had evolved at least one hearing problem. A great majority (93.5%) said that they had no hearing problems before the transplantation. Among the TX patients, 31.1% had pressure sensation in the ear, 41.3% had tinnitus, and 19.6% had dizziness. While 97.8% of the patients said they did not need help for hearing, only 2.2% stated otherwise. None of the patients had sudden hearing loss after kidney transplantation. On the other hand, 8.7% of HD patients had pressure sensation in the ear, 17.4% had tinnitus and 26.1% had dizziness. Ear ache-pressure sensation and tinnitus complaints were more common among the TX patients than HD patients (Table 1; Figure 1).

**TABLE 1:** Hearing problems in the HD and TX patients according to the survey results.

	TX *n* = 46	HD *n* = 23	*p*
Pain-Pressure sensation %	31.1	8.7	0.04
Tinnitus %	41.3	17.4	0.047
Dizziness %	19.6	26.1	0.53
Hearing loss before transplantation %	6.5	--	--
Hearing loss after transplantation %	10.9	--	--

### Laboratory Findings, Demographic and Audiometric Data

Chronic kidney disease etiologies observed in HD patients were hypertension (HT) in 21.7%, chronic glomerulonephritis (CGN) in 13.3%, unknown/other causes in 65%. In the TX group, 26.1% had HT, 6.5% had polycystic kidney disease (PKD), 10.9% had CGN, and 56.5% had unknown and other causes.

**FIGURE 2:**
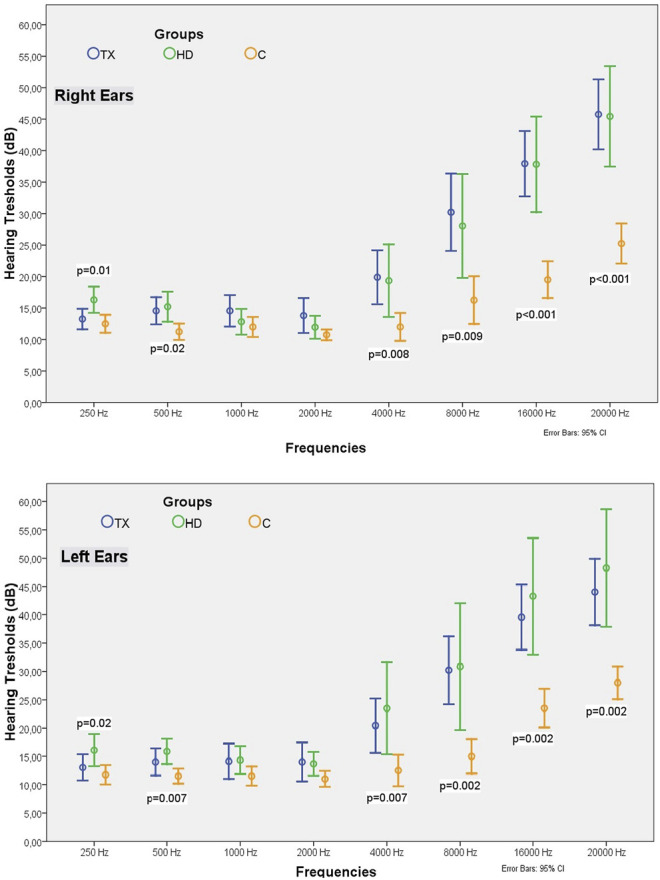
Comparison of hearing tresholds of the TX, HD and C groups in different frequencies.

The TX, HD, and C groups were similar in terms of age (36.6 ± 11.9, 35.4 ± 8.7, and 33.0 ± 10.0 years, respectively; *p* = 0.13) and gender distribution (male gender: 65.2%, 60.9%, and 70.0%, respectively; *p* = 0.82). The SBP of TX and HD patients was higher than that of healthy controls (Table 2; Figure 2). As expected, the eGFR of the C group was significantly higher than the TX group, (112.2 ± 18.0 vs. 76.3 ± 16.6 ml/min/1.73 m^2^, *p* < 0.001). Other laboratory parameters are summarised in Table 2.

**TABLE 2:** Comparison of demographic, laboratory and audiometric results of the TX, HD and C groups.

Kruskal Wallis Analysis		Tx *n* = 46	HD *n* = 23	C *n* = 20	*p*
		Mean ± SD	Mean ± SD	Mean ± SD	
Age (Year)		36.6 ± 11.9	35.4 ± 8.7	33.0 ± 10.0	0.13
Gender (Male %)		65.2	60.9	70.0	0.82
HT (%)		67.4	56.5	0	0.43
BMI (kg/m^2^)		25.5 ± 4.7	23.5 ± 6.2	25.2 ± 5.0	0.13
SBP (mmHg)		126.0 ± 14.4	131.8 ± 21.6	114.2 ± 8.4^a^	<0.001
DBP (mmHg)		78.9 ± 10.2	79.3 ± 17.2	75.7 ± 7.1	0.33
BUN (mg/dl)		16.7 ± 7.8^b^	49.8 ± 24.8^b^	12.0 ± 2.9^b^	<0.001
sCr (mg/dl)		1.15 ± 0.26^b^	8.60 ± 3.49^b^	0.81 ± 0.13^b^	<0.001
eGFR (ml/dk/1.73m^2^)		76.3 ± 16.6	-	112.2 ± 18.0	<0.001
FBG (mg/dl)		87.7 ± 10.6	90.7 ± 15.9	92.5 ± 12.1	0.29
Na (mEq/L)		139.9 ± 2.19	137.3 ± 1.91^a^	140.0 ± 2.59	<0.001
K (mEq/L)		4.35 ± 0.44	5.35 ± 0.82^a^	4.64 ± 0.67	<0.001
Ca (mg/dl)		9.70 ± 0.52	8.36 ± 0.88^a^	9.43 ± 0.33	<0.001
P (mg/dl)		3.16 ± 0.60	5.41 ± 0.94^a^	3.57 ± 0.50	<0.001
TG (mg/dl)		164.9 ± 71.0	166.9 ± 89.9	138.6 ± 72.8	0.33
LDL cholesterol (mg/dl)		118.3 ± 36.3	94.7 ± 28.7^a^	126.3 ± 39.8	0.01
UA (mg/dl)		5.9 ± 1.3	6.2 ± 1.1	5.2 ± 1.7	0.15
Albumin (gr/L)		43.3 ± 3.8^b^	37.2 ± 5.3^b^	47.6 ± 3.4^b^	<0.001
Hemoglobin (gr/dl)		14.1 ± 1.8	10.6 ± 1.4^a^	14.9 ± 1.8	<0.001
Odiometric data					
Right Ear	250 Hz (dB)	13.26 ± 5.49	16.30 ± 4.81^a^	12.50 ± 3.03	0.01
	500 Hz (dB)	14.56 ± 7.28	15.21 ± 5.53	11.25 ± 2.75^a^	0.02
	1,000 Hz (dB)	14.56 ± 8.42	12.82 ± 4.72	12.00 ± 3.40	0.60
	2,000 Hz (dB)	13.80 ± 9.32	11.95 ± 4.19	10.75 ± 1.83	0.69
	4,000 Hz (dB)	19.89 ± 14.43	19.34 ± 13.34	12.00 ± 4.70^a^	0.008
	8,000 Hz (dB)	30.21 ± 20.65	28.04 ± 19.05	16.25 ± 8.09^a^	0.009
	16,000 Hz (dB)	37.93 ± 17.49	37.82 ± 17.56	19.50 ± 6.26^a^	<0.001
	20,000 Hz (dB)	45.76 ± 18.70	45.43 ± 18.45	25.25 ± 6.78^a^	<0.001
Left Ear	250 Hz (dB)	13.04 ± 7.78	16.08 ± 6.56^a^	11.75 ± 3.72	0.02
	500 Hz (dB)	14.02 ± 8.07	15.86 ± 5.14	11.50 ± 2.85^a^	0.007
	1,000 Hz (dB)	14.13 ± 10.50	14.34 ± 5.70	11.50 ± 3.66	0.16
	2,000 Hz (dB)	14.02 ± 11.62	13.69 ± 4.81	11.00 ± 3.07	0.18
	4,000 Hz (dB)	20.43 ± 16.22	23.47 ± 18.79	12.50 ± 5.96^a^	0.007
	8,000 Hz (dB)	30.21 ± 20.24	30.86 ± 25.87	15.00 ± 6.48^a^	0.002
	16,000 Hz (dB)	39.56 ± 19.34	43.26 ± 23.81	23.50 ± 7.27^a^	0.002
	20,000 Hz (dB)	44.02 ± 19.79	48.26 ± 14.05	28.25 ± 6.34^a^	0.002
Right Ear SRT (dB)		14.13 ± 5.50	13.47 ± 4.37	11.25 ± 2.75^a^	0.04
Left Ear SRT (dB)		14.02 ± 7.19	14.34 ± 4.07	11.00 ± 2.05^a^	0.01
Right Ear SD (%)		95.28 ± 9.31	95.47 ± 3.90	99.00 ± 1.77^a^	0.005
Left Ear SD (%)		95.41 ± 9.24	95.47 ± 3.90	98.80 ± 2.28^a^	0.009

a It represents the group whose value is different from the other two groups.

b It states that the values of each three groups are different from each other.

The hearing thresholds at 250 Hz in both ears of HD patients were significantly higher than that of TX and C group patients (right ear measurements of the HD, TX, C groups were 16.30 ± 4.81 vs. 13.26 ± 5.49, 12.50 ± 3.03 dB, respectively; *p* = 0.01) (Table 2; Figure 2).

In both ears, the hearing thresholds in the TX and HD groups at 500 Hz were similar and significantly higher than that in the C group (right ear measurements of the HD, TX, C groups were 15.21 ± 5.53, 14.56 ± 7.28 vs. 11.25 ± 2.75 dB, respectively; *p* = 0.02) (Table 2; Figure 2).

Hearing thresholds in all groups were similar between the 1,000–2,000 Hz range (*p* > 0.05 for all).

At all frequencies between 4,000–20,000 Hz, the hearing thresholds of the HD and TX groups were similar in both ears and were significantly higher than that in the C group. As the frequency increased from 4,000 Hz to 20,000 Hz, the hearing thresholds of HD and TX patients also increased (*p* < 0.01 for all) (Table 2; Figure 2).

The first test of immittance testing was tympanometry, which returned normal values for middle ear pressure levels in all patients. The second test was the acoustic reflex test, which revealed that the stapedius reflexes were bilaterally normal for all participants.

For both ears, the SRT values of the HD and TX groups were significantly higher than that of the C group (right ear measurements of the TX, HD, C groups were 14.13 ± 5.50, 13.47 ± 4.37 and 11.25 ± 2.75 dB, respectively; *p* = 0.04) (Table 2). The SD values in the HD and TX groups were significantly lower in both ears than the C group (right ear measurements of the TX, HD, C groups were, 95.28 ± 9.31, 95.47 ± 3.90 and 99.00 ± 1.77, respectively; *p* = 0.005) (Table 2).

### Hearing Loss Rates

**FIGURE 3:**
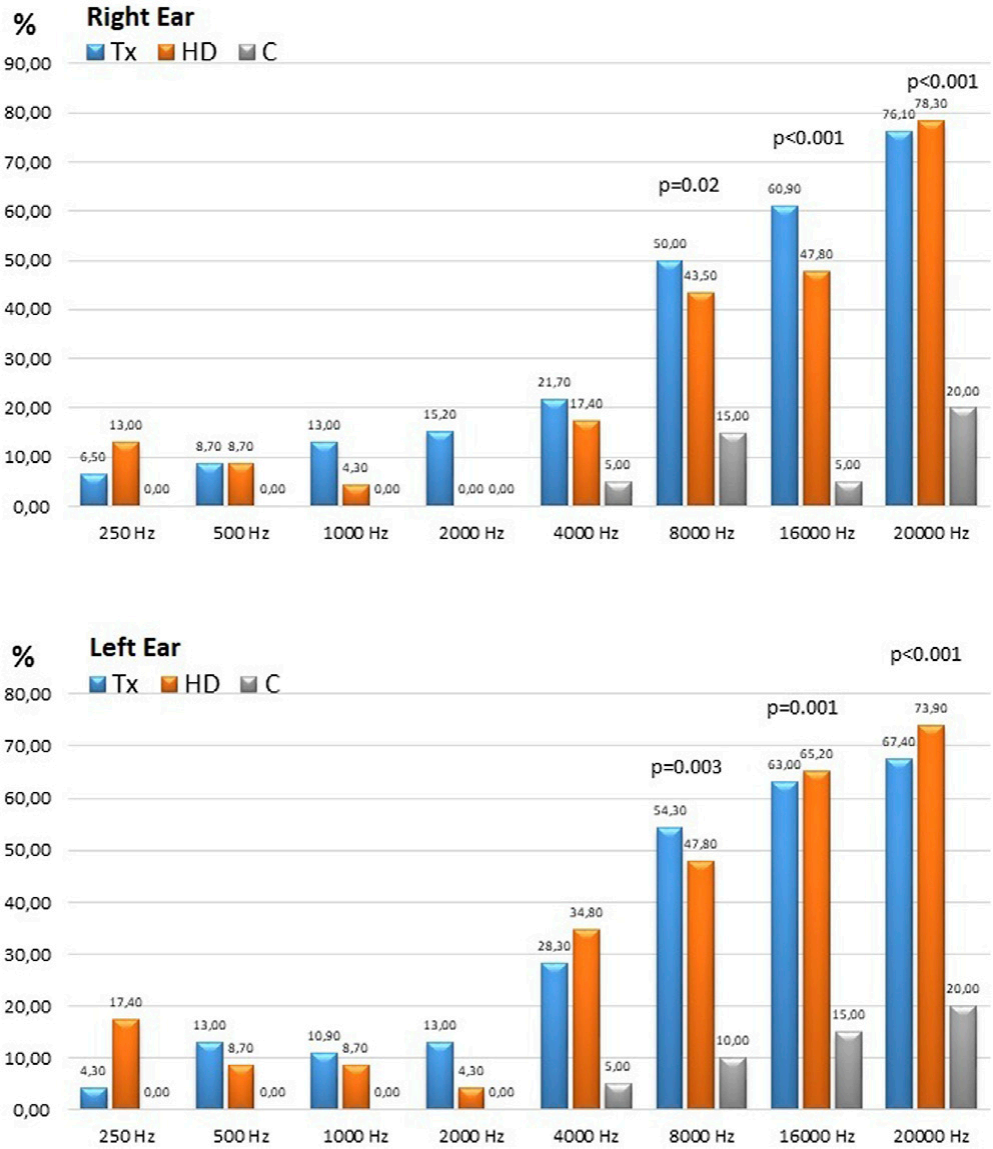
The percentages of the patients with hearing defects at different frequencies in the HD, TX and C groups.

The normal hearing thresholds was accepted as 20 dB for the 250–8,000 Hz frequency range and 30 dB for the 16,000 and 20,000 Hz frequency range. The percentage of patients with hearing defects in all are given in Table 3. As the frequency increased in the TX and HD groups, the proportion of patients with hearing impairment also increased, reaching 76% and 78% at 20,000 Hz. The percentage of patients with hearing loss at frequencies of 8,000 Hz and above was similar in the TX and HD groups and significantly higher than in the C group (Figure 3).

**TABLE 3:** The percentages of the patients with hearing defects at different frequencies in HD, TX and C groups.

	Hearing impairement treshold (dB)	Frequency (Hz)	Tx *n* = 46 (%)	HD *n* = 20 (%)	C *n* = 20 (%)	*p*
Right Ear	20	250	6.5	13	0	0.23
		500	8.7	8.7	0	0.39
		1,000	13	4.3	0	0.14
		2,000	15.2	0	0	0.03
		4,000	21.7	17.4	5	0.24
		8,000	50	43.5	15^a^	0.02
	30	16,000	60.9	47.8	5^a^	<0.001
		20,000	76.1	78.3	20^a^	<0.001
Left Ear	20	250	4.3	17.4	0	0.05
		500	13	8.7	0	0.23
		1,000	10.9	8.7	0	0.31
		2,000	13	4.3	0	0.14
		4,000	28.3	34.8	5	0.057
		8,000	54.3	47.8	10^a^	0.003
	30	16,000	63	65.2	15^a^	0.001
		20,000	67.4	73.9	20^a^	<0.001

a It represents the group whose value is different from the other two groups.

### Comparison of TX Patients Using Tacrolimus and mTOR Inhibitors

**FIGURE 4:**
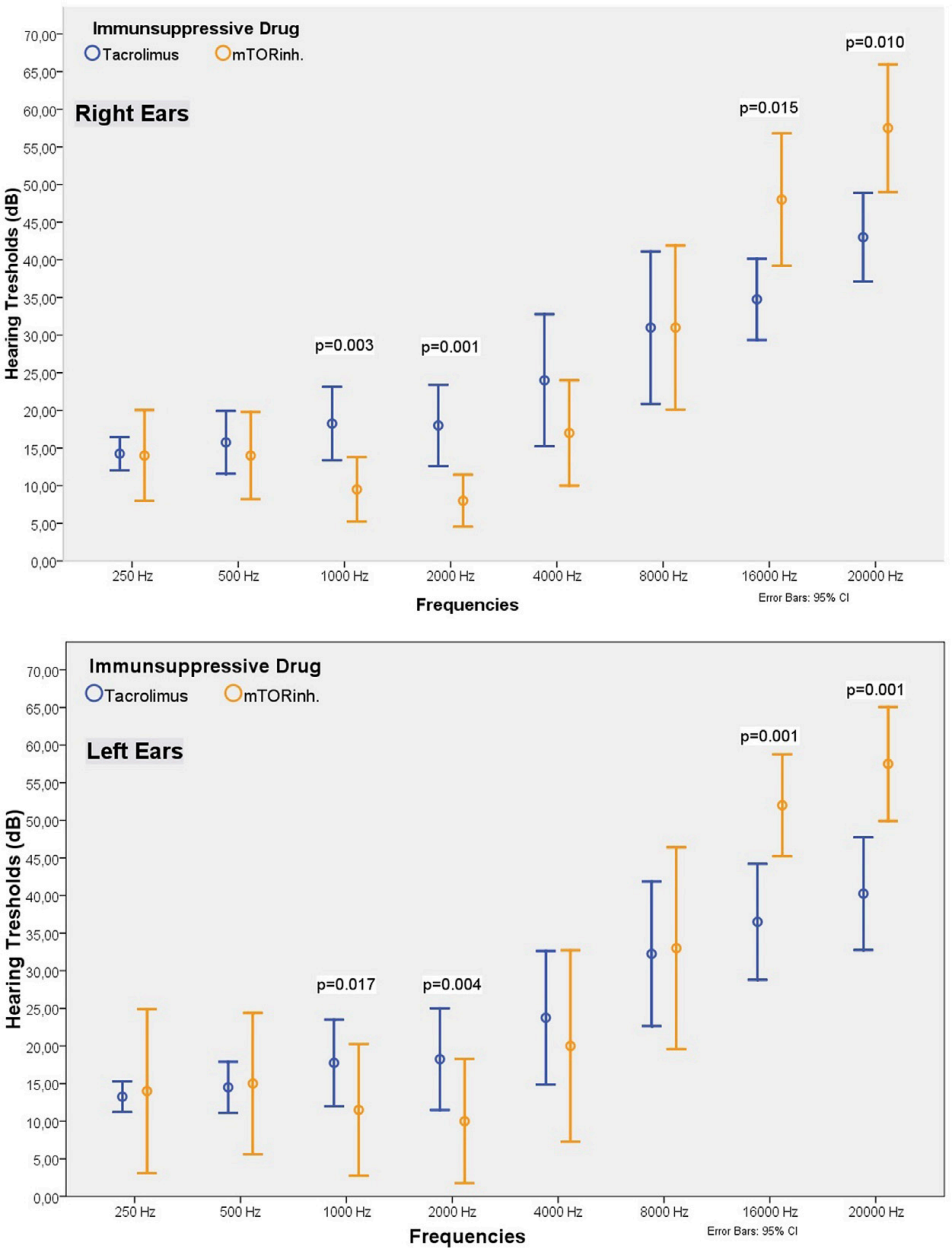
Comparison of the hearing threshold values of patients receiving tacrolimus or an mTOR inhibitor (matched groups).

Among our cohort, 30 patients used tacrolimus and 16 used mTOR inhibitor. There were no differences in terms of hearing threshold between the two groups within 250–500 Hz frequency range. In both ears, the hearing threshold at 1,000–2,000 Hz in patients receiving tacrolimus was significantly higher than the value among the patients receiving an mTOR inhibitor (Table 4; Figure 4). The hearing thresholds at high frequencies such as 16,000 and 20,000 Hz in patients using an mTOR inhibitor were significantly higher for both ears than the patients using tacrolimus (49.06 ± 13.44 vs. 32.00 ± 16.64 dB at 16,000 Hz, *p* = 0.001; and 58.43 ± 12.74 dB vs. 39.00 ± 17.97 dB at 20,000 Hz for the right ear; *p* < 0.001) (Table 4; Figure 4). However, the mean age of the patients in the group receiving an mTOR inhibitor was higher than the group who received tacrolimus (47.81 ± 11.47 vs. 33.70 ± 9.14 years, *p* < 0.001), had passed a longer time after transplantation (98.50 ± 51.16 vs. 62.93 ± 35.14 months, *p* = 0.02) and had lower eGFR (62.98 ± 20.20 vs. 79.96 ± 16.50 ml/min/1.73 m^2^, *p* = 0.001). Since these parameters are known to affect hearing, when the analysis was repeated after excluding some patients so that the two groups were matched in terms of age, eGFR and the time elapsed after kidney transplantation, it was observed that the hearing thresholds at 1,000–2,000 Hz in the patients receiving tacrolimus continued to be significantly higher than those receiving an mTOR inhibitor, and the hearing thresholds at 16,000–20,000 Hz in the patients who received an mTOR inhibitor were still higher than the patients who received tacrolimus (48.00 ± 12.29 vs. 34.75 ± 11.52 dB at 16,000 Hz, *p* = 0.015 and 57.50 ± 11.84 vs. 43.00 ± 12.60 dB at 20,000 Hz for the right ear, *p* < 0.001) (Table 4; Figure 4).

**TABLE 4:** Comparison of the demographic, laboratory and audiometric findings of the TX patients using tacrolimus or an mTOR inhibitor.

Mann Whitney U		Non matched			Matched for age, eGFR and post Tx time		
		Tacrolimus *n* = 30	mTOR *n* = 16	*p*	Tacrolimus *n* = 20	mTOR *n* = 10	*p*
		Mean ± SD	Mean ± SD		Mean ± SD	Mean ± SD	
Age (Year)		33.70 ± 9.14	47.81 ± 11.47	<0.001	37.45 ± 8.01	41.6 ± 8.9	0.35
Gender (Male %)		63.3	68.8	0.71	65.0	70.0	1.00
PostTX time (months)		62.93 ± 35.14	98.50 ± 51.16	0.02	67.00 ± 32.37	93.50 ± 46.79	0.14
HT (%)		73.3	56.2	0.32	85.0	60.0	0.18
Pre TX RRT time		26.86 ± 51.57	55.06 ± 92.54	0.17	30.05 ± 60.37	72.20 ± 113.79	0.16
BMI (kg/m^2^)		25.95 ± 4.99	24.71 ± 4.15	0.41	26.18 ± 4.39	23.95 ± 4.28	0.18
SBP(mmHg)		125.13 ± 15.16	127.87 ± 13.19	0.42	126 ± 16.93	126.81 ± 13.25	0.57
DBP(mmHg)		75.30 ± 8.87	85.87 ± 9.30	0.002	74.65 ± 9.64	86.70 ± 11.32	0.02
BUN (mg/dl)		13.10 ± 3.71	23.68 ± 9.05	<0.001	13.65 ± 3.92	23.60 ± 9.00	0.002
sCr (mg/dl)		1.07 ± 0.22	1.29 ± 0.27	0.012	1.12 ± 0.21	1.22 ± 0.28	0.28
eGFR ml/min/1.73m^2^		79.96 ± 16.50	62.98 ± 20.20	0.001	72.35 ± 10.42	72.80 ± 18.63	0.27
FBG (mg/dl)		89.13 ± 11.58	85.12 ± 8.26	0.35	92.75 ± 11.76	85.50 ± 8.97	0.23
Na (mEq/L)		139.50 ± 1.83	140.66 ± 2.63	0.16	139.60 ± 1.60	140.07 ± 2.09	0.94
K (mEq/L)		4.36 ± 0.41	4.33 ± 0.50	0.90	4.36 ± 0.43	4.33 ± 0.41	0.96
Ca (mg/dl)		9.71 ± 0.46	9.68 ± 0.64	0.77	9.75 ± 0.44	9.80 ± 0.56	0.67
P (mg/dl)		3.17 ± 0.65	3.15 ± 0.51	0.65	3.04 ± 0.65	3.25 ± 0.62	0.58
TG (mg/dl)		166.00 ± 72.98	163.06 ± 69.68	0.86	172.70 ± 67.68	157.40 ± 74.55	0.45
LDL cholesterol (mg/dl)		105.13 ± 27.53	148.69 ± 36.79	<0.001	107.70 ± 27.04	147.77 ± 31.20	0.006
UA (mg/dl)		5.74 ± 1.19	6.38 ± 1.42	0.15	5.93 ± 1.16	6.57 ± 1.65	0.27
Albumin (gr/L)		44.62 ± 3.42	40.67 ± 3.24	0.001	44.59 ± 3.81	41.11 ± 3.50	0.017
Hemoglobin (gr/dl)		14.56 ± 1.95	13.48 ± 1.60	0.07	14.64 ± 2.00	13.44 ± 1.84	0.16
Tacrolimus C0 levels (mcg/L)		5.28	-	-	5.35 ± 1.77	-	-
Everolimus C0 levels (mcg/L)		-	3.49	-	-	3.94 ± 1.68	-
Odiometric data						
Right Ear	250Hz (dB)	13.33 ± 4.22	13.12 ± 7.50	0.72	14.25 ± 4.66	14.00 ± 8.43	0.71
	500Hz (dB)	14.83 ± 7.59	14.06 ± 6.88	0.98	15.75 ± 8.92	14.00 ± 8.09	0.76
	1000 Hz (dB)	16.33 ± 9.27	11.25 ± 5.32	0.046	18.25 ± 10.42	9.50 ± 5.98	0.003
	2000 Hz (dB)	15.50 ± 10.03	10.62 ± 7.04	0.016	18.00 ± 11.51	8.00 ± 4.83	0.001
	4000 Hz (dB)	20.33 ± 16.18	19.06 ± 10.83	0.81	24.00 ± 18.75	17.00 ± 9.77	0.39
	8000 Hz (dB)	27.83 ± 21.64	34.68 ± 18.48	0.11	31.00 ± 21.61	31.00 ± 15.23	0.62
	16000 Hz (dB)	32.00 ± 16.64	49.06 ± 13.44	0.001	34.75 ± 11.52	48.00 ± 12.29	0.015
	20000 Hz (dB)	39.00 ± 17.97	58.43 ± 12.74	<0.001	43.00 ± 12.60	57.50 ± 11.84	0.010
Left Ear	250Hz (dB)	12.66 ± 3.88	13.75 ± 12.31	0.37	13.25 ± 4.37	14.00 ± 15.23	0.14
	500Hz (dB)	13.83 ± 6.52	14.37 ± 10.62	0.95	14.50 ± 7.23	15.00 ± 13.12	0.56
	1000 Hz (dB)	15.83 ± 10.51	10.93 ± 10.03	0.004	17.75 ± 12.29	11.50 ± 12.25	0.017
	2000 Hz (dB)	16.00 ± 12.27	10.31 ± 9.56	0.004	18.25 ± 14.44	10.00 ± 11.54	0.004
	4000 Hz (dB)	20.66 ± 16.28	20.00 ± 16.63	0.68	23.75 ± 18.97	20.00 ± 17.75	0.44
	8000 Hz (dB)	28.66 ± 21.12	33.12 ± 18.78	0.26	32.25 ± 20.55	33.00 ± 18.73	0.61
	16000 Hz (dB)	32.83 ± 18.78	52.18 ± 13.41	<0.001	36.50 ± 16.47	52.00 ± 9.48	0.001
	20000 Hz (dB)	36.83 ± 18.40	57.50 ± 14.94	<0.001	40.25 ± 16.01	57.50 ± 10.60	0.001
Right Ear SRT (dB)		14.50 ± 5.92	13.43 ± 4.73	0.73	15.50 ± 6.66	13.00 ± 5.37	0.29
Left Ear SRT (dB)		13.83 ± 5.20	14.37 ± 10.14	0.50	14.50 ± 5.82	14.50 ± 12.79	0.13
Right Ear SD (%)		93.66 ± 11.06	98.31 ± 2.91	0.026	91.90 ± 13.11	98.10 ± 3.41	0.04
Left Ear SD (%)		93.80 ± 11.02	98.43 ± 2.55	0.022	92.10 ± 13.08	98.30 ± 2.90	0.03

SRT values for both ears were not different between the mTOR inhibitor and tacrolimus-receiving groups (*p* > 0.05). However, patients who received an mTOR inhibitor had higher SD values in both ears than the patients who received tacrolimus (right ear measurements were 98.31 ± 2.91 and 93.66 ± 11.06 respectively, *p* = 0.026). When both groups were matched, the mean SD value of the patients receiving mTOR inh was still higher than those receiving tacrolimus (Table 4; Figure 4).

In the TX group, the hearing threshold was strongly correlated with age at frequencies of 8,000 Hz and above (*p* = 0.002 and, r = 0.44 at 16,000 Hz, and *p* = 0.001, r = 0.46 at 20,000 Hz). The hearing threshold was inversely correlated with eGFR and serum *p* values (Table 5). No correlation was found between blood tacrolimus or mTOR inhibitor levels and hearing thresholds. In the HD patients, the correlation between hearing thresholds and age started at 4,000 Hz and continued up to 20,000 Hz (*p* = 0.03, r = 0.45 for 16,000 Hz, and *p* = 0.05, r = 0.39 for 20,000 Hz) (Table 5).

**TABLE 5:** Correlation of hearing thresholds with clinical parameters (right ear data only shown).

	Frequency		Age	Post Tx time	eGFR	Ca	P	HD vintage	kt/v	URR	Tacrolimus levels C0 (*n* = 30)	mTOR inhibitor levels C0 (*n* = 16)
TX (n = 46)	Right 16,000 Hz	*p*	0.002	0.055	0.008	0.85	0.014	NA	NA	NA	0.50	0.98
		r	0.44	0.28	−0.38	0.02	−0.36	NA	NA	NA	−0.12	0.005
	Right 20,000 Hz	*p*	0.001	0.052	0.008	0.78	0.013	NA	NA	NA	0.50	0.98
		r	0.46	0.28	−0.38	0.04	−0.36	NA	NA	NA	−0.12	0.004
HD (n = 23)	Right 16,000 Hz	*p*	0.03	NA	NA	0.61	0.4	0.040	0.31	0.55	NA	NA
		r	0.45	NA	NA	0.11	0.18	0.44	0.22	0.13	NA	NA
	Right 20,000 Hz	*p*	0.05	NA	NA	0.51	0.45	0.043	0.35	0.61	NA	NA
		r	0.39	NA	NA	0.14	0.16	0.43	0.20	0.11	NA	NA
C (n = 20)	Right 16,000 Hz	*p*	0.048	NA	0.98	0.36	0.41	NA	NA	NA	NA	NA
		r	0.44	NA	0.006	0.21	−0.20	NA	NA	NA	NA	NA
	Right 20,000 Hz	*p*	0.30	NA	0.99	0.37	0.26	NA	NA	NA	NA	NA
		r	0.24	NA	0.001	0.21	−0.27	NA	NA	NA	NA	NA

NA, not applicable.

## Discussion

This study is the first to evaluate the hearing function of kidney transplant patients with EHFA and evaluate the effect of immunosuppressive agents on hearing. Our main findings can be summarized as follows. The majority of kidney transplant patients have hearing-related abnormalities. Kidney transplant patients have hearing loss that does not affect the middle frequencies but is evident at high frequencies, and their hearing level is as bad as HD patients. Hearing loss in CKD patients is likely to be permanent and a kidney transplant may not improve their hearing problems. The SRT and SD values were impaired with hearing loss. The use of tacrolimus seems to cause auditory deficit in the 1,000–2,000 Hz range, and the use of mTOR inhibitor mostly at high frequencies such as 16,000 and 20,000 Hz.

The frequency of sensorineural hearing loss in CKD patients ranges from 28 to 77%, and hearing function declines as the stage of CKD increases (12–14). The condition has a high prevalence in HD patients and is often bilateral (14). It has been reported that the frequency of hearing loss increases as the total duration of renal disease and HD duration increases with age advancement (14–16). Although many studies have shown that hearing loss is more pronounced at higher frequencies in HD patients (14, 15), few studies have suggested that it does not differ from low to high frequencies or is more pronounced at low frequencies (17, 18). In HD patients, there are many known risk factors to explain sensorineural hearing loss, such as the use of ototoxic drugs like furosemide, the presence of DM and advanced age. Apart from these classical risk factors, many pathogenetic mechanisms such as the direct effects of uremia itself, development of hydrops in the endolymph fluid of the inner ear, changes in endolymph composition and electrolyte imbalances, aluminium deposition and dialysis amyloid deposition have been blamed (12).

Unfortunately, there is not sufficient evidence that these abnormalities improve after kidney transplantation. In two studies conducted by Mitschke et al in 1975 and 1977, it was suggested that audiometric abnormalities returned to normal after kidney transplantation. The authors showed that 7 of 10 HD patients’ hearing thresholds within the range of 256–8192 Hz returned to normal after kidney transplantation. The three patients in whom the hearing defect did not improve had hereditary nephritis (19). In the second study, it was shown that the hearing thresholds among 13 HD patients within the 2,000–8,000 Hz range returned to normal at an average of 21 months after kidney transplantation (from 29.3 to 7.7 for 8,000 Hz) (20). However, the pre-transplant serum BUN (102 mg/dl), creatinine (14 and 14.6 mg/dl) and albumin (2.7 g/dl) values of the patients in these two studies were below today’s standards, indicating insufficient dialysis. In addition, the fact that the patients included in the study used ototoxic drugs such as digital and aluminium compounds and gentamicin necessitates a cautious approach to the results of these two studies. In our study, we showed that the proportion of transplant patients with hearing defects increased as the frequency went from 4,000 to 20,000 Hz, similar to HD patients, and the hearing thresholds increased as the frequency increased. In other words, the frequency of hearing defect and the hearing thresholds in kidney transplant patients were as bad as HD patients. De Los Santos et al. performed audiometric evaluation of 45 HD patients, 43 TX patients, and 40 healthy individuals, and showed that the prevalance of mild hearing loss at 3,000 Hz and was higher among the TX patients than the HD patients (21). However, there is no information about the immunosuppressive regimen and drug blood levels used in this study. Bains et al. evaluated the cochlear function abnormalities of stage 3–5 CKD patients and healthy controls with pure-tone audiometry and BERA [Brainstem Evoked Response Audiometry-BERA is an objective and non-invasive method for assessing the auditory pathways from the auditory nerve to the brainstem]. They showed that the hearing thresholds among the CKD patients were higher than healthy controls at all frequencies between 250–8,000 Hz, especially at higher frequencies (22). However, there was no significant improvement in the hearing thresholds of Stage 5 CKD patients 1 year after kidney transplantation compared to pre-transplant levels. When the same patients were evaluated with BERA, the researchers showed that CKD patients had more absolute and interpeak delays of waves I, III, and V than healthy controls. After kidney transplantation, there was only some improvement in the absolute delays of waves I, III, and V, but no significant improvement in interpeak delays. In the BERA test, absolute peak delay cannot distinguish the hearing losses from cochlea or post-cochlear auditory pathways (23). However, interpeak delays are not affected by cochlear function and reflect the defect between the central pathways of hearing (23). Lack of improvement in interpeak delays may indicate a problem with the auditory nerve. According to the results of these two studies as well as our study, it is possible to conclude that the majority of TX patients have hearing defects that cannot be noticed by patients, this defect is more prominent especially at high frequencies, and there is no significant improvement in hearing defect after kidney transplantation. There are two possible reasons for this. The first one is permanent damage to the cochlea from the CKD process: The data showing that the damage may be permanent in these patients come from a very old study conducted by Oda et al. In the pathological examination of the temporal bones of eight patients who died due to various reasons after kidney transplantation, the authors have shown that there was significant damage and even loss of the Corti organ, the petrification of the stria vascularis, especially in patients who had long-term dialysis treatment and had multiple kidney transplants (24). The second reason is the neurotoxic effects of immunosuppressive drugs on the auditory nerve. Calcineurin inhibitors are neurotoxic drugs. Ototoxicity could be a manifestation of neurotoxicity associated with CNI use and may not be noticed by the patient, but can be demonstrated by audiometric tests. Case reports of sudden hearing loss after kidney transplantation are available in the literature (25–28). Gulleroglu et al. (25) reported significant hearing loss at 4,000–8,000 Hz frequency in two pediatric kidney transplant patients, while tacrolimus levels were as high as 22 and 29 nmol/L in both patients. Even when drug level was reduced, the progression of the hearing loss stopped but did not improve. The same author later found hearing loss between 4,000 and 8,000 Hz by pure-tone audiometry in 17 of 27 pediatric kidney transplant patients and showed that patients with hearing loss had higher CsA levels than those without hearing loss (29). In our study, we could not demonstrate a relationship between blood tacrolimus or mTOR inhibitor levels and hearing thresholds. It is known that hearing problems occur in other patient groups who have not been exposed to uremia for a long time, or after organ transplantations other than kidney transplantation, or in other patient groups who have to use CNI due to glomerulonephritis. Rifai et al. reported that CNI levels were very high in five patients, who developed sudden hearing loss after orthotopic liver transplantation, although there was no other risk factor, and hearing loss was permanent in four of these patients, even though the drug level was reduced to the normal range (30). It was also reported that 35% of the patients who used tacrolimus rather than cyclosporine had various hearing problems (31). In their 2012 study, Rifai et al. (32) detected hearing loss with pure tone audiometry in 53% of 70 liver transplant patients. Half of the patients who did not describe any hearing problems had audiometric abnormalities. Interestingly, in our study, patients receiving an mTOR inhibitor had worse hearing thresholds and SD values at 16,000 and 20,000 Hz compared to patients receiving tacrolimus. This finding may be difficult to explain, as no major neurotoxicity of mTOR inhibitors has been demonstrated (7–9). However, the mTOR pathway plays a role in axonal sprouting, astrocyte metabolism, mitochondrial functions, axonal regeneration and myelination, regulation of synaptic activity, and perhaps most importantly, the expression of some ion channels and receptors (9). In the realization of hearing, ion channels and receptors in hair cells play a key role in the conversion of mechanical energy into electrical messages by hair cells in the cochlea. mTOR inhibitors may be causing damage at the cochlea level. On the other hand, patients using tacrolimus had worse hearing thresholds at frequencies of 1,000–2,000 Hz than patients using an mTOR inhibitor. Tacrolimus is a neurotoxic drug and a defect at 1,000–2,000 Hz may be a sign of neurotoxicity.

The SRT values were higher while the SD values were lower for the study groups than the control group. These results are in close agreement with pure tone threshold results and confirm the validity of the pure tone thresholds. The immittance testing returned normal results in all groups. This may be expected since HD, kidney transplantation or the drugs used had no effects on the middle ear pressure and acoustic reflex.

Our study had some limitations. Since it was conducted in two different locations, the measurements made by two audiologists may partially affect the results. Its cross-sectional design rather than being a prospective one may be another limitation. However, there are only three studies in the literature evaluating hearing problems in kidney transplant patients with audiometry, while few others have evaluated the condition using questionnaires only. Our study is the first of its kind to make analyses with EHFA. In addition, there is no other study in the literature that evaluates the relationship between hearing problems and the immunosuppressive drug type used. The number of patients in our study was determined by power analysis, and the power of our study was over 80%.

In conclusion, there are defects in hearing and cochlear functions in kidney transplant patients due to permanent hearing defects because of CKD and the additive effects of immunosuppressive drugs. Hearing defects probably do not improve after a kidney transplant. This issue needs to be investigated with prospective studies.

## Data Availability

The raw data supporting the conclusions of this article will be made available by the authors, without undue reservation.
